# The Brainbox*—*a tool to facilitate correlation of brain magnetic resonance imaging features to histopathology

**DOI:** 10.1093/braincomms/fcad307

**Published:** 2023-11-08

**Authors:** Wolfgang Faigle, Marco Piccirelli, Tibor Hortobágyi, Karl Frontzek, Amelia Elaine Cannon, Wolfgang Emanuel Zürrer, Tobias Granberg, Zsolt Kulcsar, Thomas Ludersdorfer, Katrin B M Frauenknecht, Regina Reimann, Benjamin Victor Ineichen

**Affiliations:** 1 Neuroimmunology and MS Research Section, Neurology Clinic, University Zurich, University Hospital Zurich, CH-8091 Zurich, Switzerland; Department of Neuroradiology, Clinical Neuroscience Center, University Hospital Zurich, University of Zurich, CH-8091 Zurich, Switzerland; Institute of Neuropathology, University of Zurich, CH-8091 Zurich, Switzerland; Institute of Neuropathology, University of Zurich, CH-8091 Zurich, Switzerland; Queen Square Brain Bank for Neurological Disorders, UCL Queen Square Institute of Neurology, WC1N 1PJ London, United Kingdom; Department of Neuroradiology, Clinical Neuroscience Center, University Hospital Zurich, University of Zurich, CH-8091 Zurich, Switzerland; Department of Neuroradiology, Clinical Neuroscience Center, University Hospital Zurich, University of Zurich, CH-8091 Zurich, Switzerland; Department of Neuroradiology, Karolinska University Hospital, S-141 86 Stockholm, Sweden; Department of Neuroradiology, Clinical Neuroscience Center, University Hospital Zurich, University of Zurich, CH-8091 Zurich, Switzerland; 1 Neuroimmunology and MS Research Section, Neurology Clinic, University Zurich, University Hospital Zurich, CH-8091 Zurich, Switzerland; Institute of Neuropathology, University of Zurich, CH-8091 Zurich, Switzerland; Luxembourg Center of Neuropathology (LCNP), Laboratoire National de Santé, 3555 Dudelange, Luxembourg; National Center of Pathology (NCP), Laboratoire National de Santé, 3555 Dudelange, Luxembourg; Institute of Neuropathology, University of Zurich, CH-8091 Zurich, Switzerland; Department of Neuroradiology, Clinical Neuroscience Center, University Hospital Zurich, University of Zurich, CH-8091 Zurich, Switzerland; Center for Reproducible Science, University of Zurich, CH-8001 Zurich, Switzerland

**Keywords:** neuroimaging, magnetic resonance imaging, histopathology, correlation, multiple sclerosis

## Abstract

Magnetic resonance imaging (MRI) has limitations in identifying underlying tissue pathology, which is relevant for neurological diseases such as multiple sclerosis, stroke or brain tumours. However, there are no standardized methods for correlating MRI features with histopathology. Thus, here we aimed to develop and validate a tool that can facilitate the correlation of brain MRI features to corresponding histopathology. For this, we designed the Brainbox, a waterproof and MRI-compatible 3D printed container with an integrated 3D coordinate system. We used the Brainbox to acquire post-mortem *ex vivo* MRI of eight human brains, fresh and formalin-fixed, and correlated focal imaging features to histopathology using the built-in 3D coordinate system. With its built-in 3D coordinate system, the Brainbox allowed correlation of MRI features to corresponding tissue substrates. The Brainbox was used to correlate different MR image features of interest to the respective tissue substrate, including normal anatomical structures such as the hippocampus or perivascular spaces, as well as a lacunar stroke. Brain volume decreased upon fixation by 7% (*P* = 0.01). The Brainbox enabled degassing of specimens before scanning, reducing susceptibility artefacts and minimizing bulk motion during scanning. In conclusion, our proof-of-principle experiments demonstrate the usability of the Brainbox, which can contribute to improving the specificity of MRI and the standardization of the correlation between post-mortem *ex vivo* human brain MRI and histopathology. Brainboxes are available upon request from our institution.

## Teaser

Faigle *et al.* introduces the Brainbox, a waterproof and fully MRI-compatible container with an imprinted 3D coordinate system enabling whole-brain *ex vivo* imaging for efficient MRI-histopathology correlation. The Brainbox has been validated on eight human brains. Brainboxes are available upon request from our institution.

## Graphical Abstract

**Graphical abstract:**
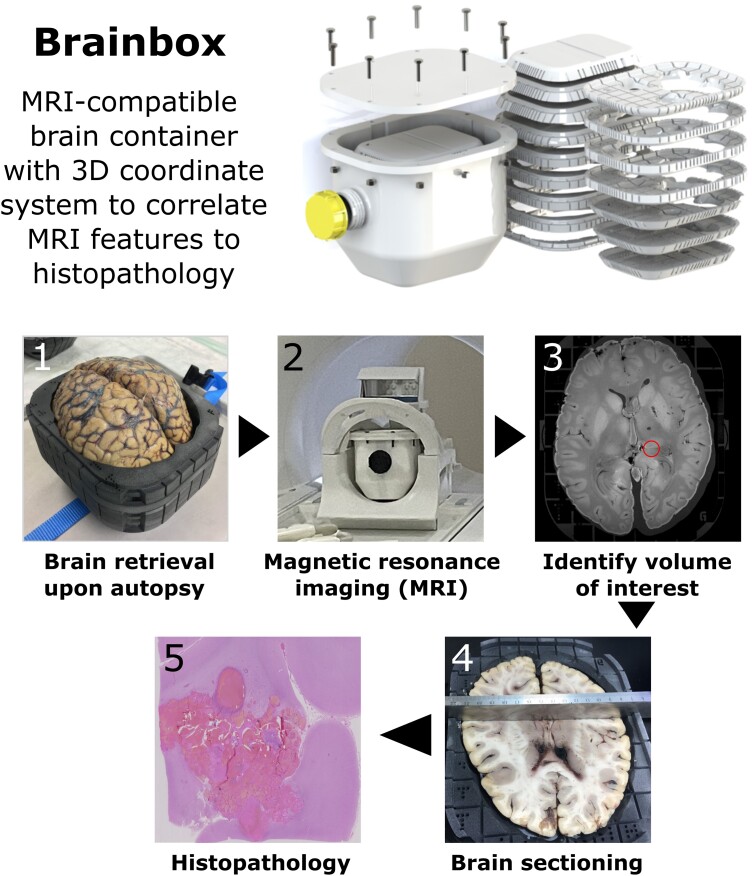

## Introduction

Brain magnetic resonance imaging (MRI) is a sensitive tool that can detect even the smallest pathological changes, making it a critical tool for the diagnosis and monitoring of various neurological diseases such as multiple sclerosis (MS), stroke and brain tumours. However, conventional MRI has limited specificity for ongoing tissue pathology. This applies particularly to neurological diseases with a complex underlying tissue pathology like MS,^1^ including breakdown of the blood-brain barrier (BBB), infiltration of macrophages, T and B lymphocytes, destruction of myelin sheaths, microglial and astrocytic activation and neurodegeneration.^2,3^ In contrast to this heterogeneous pathology, MS lesions mostly present homogeneously as hyperintense on T2-weighted images and hypointense on T1-weighted images.^4^

To gain a more comprehensive understanding of the relationship between imaging features and corresponding pathologic tissue alterations, there has been an increasing use of the approach of acquiring both MRI and histopathology within the same tissue.^5^ This approach allows for the correlation of imaging features with the corresponding tissue pathology, e.g. for MS,^6–8^ cerebral microbleeds,^9,10^ stroke,^11^ brain tumours,^12^ but also for anatomical features such as the entorhinal cortex.^13^ In addition, this allows the correlation of dedicated MRI sequences to key brain metabolites such as iron.^14,15^ Large endeavours such as the Digital Brain Bank are taking up this opportunity.^5^

The standard approach to assess the tissue signature of MRI features is manual correlation,^16–18^ which can be highly labour-intensive and is prone to intra/inter-rater variability and inaccuracy. A more standardized and accurate approach is needed to facilitate the process of MRI-histopathology correlation, particularly when correlating small imaging features such as enlarged perivascular spaces (EPVS)^19–21^ or MS lesions to their tissue signature in a 3D imaging volume.

Therefore, the objective of this study was to develop and validate a tool that facilitates MRI-histopathology correlation, enabling standardized pipelines for correlating imaging features with corresponding pathology at the tissue level.

## Materials and methods

### Development of the Brainbox

**Figure 1:**
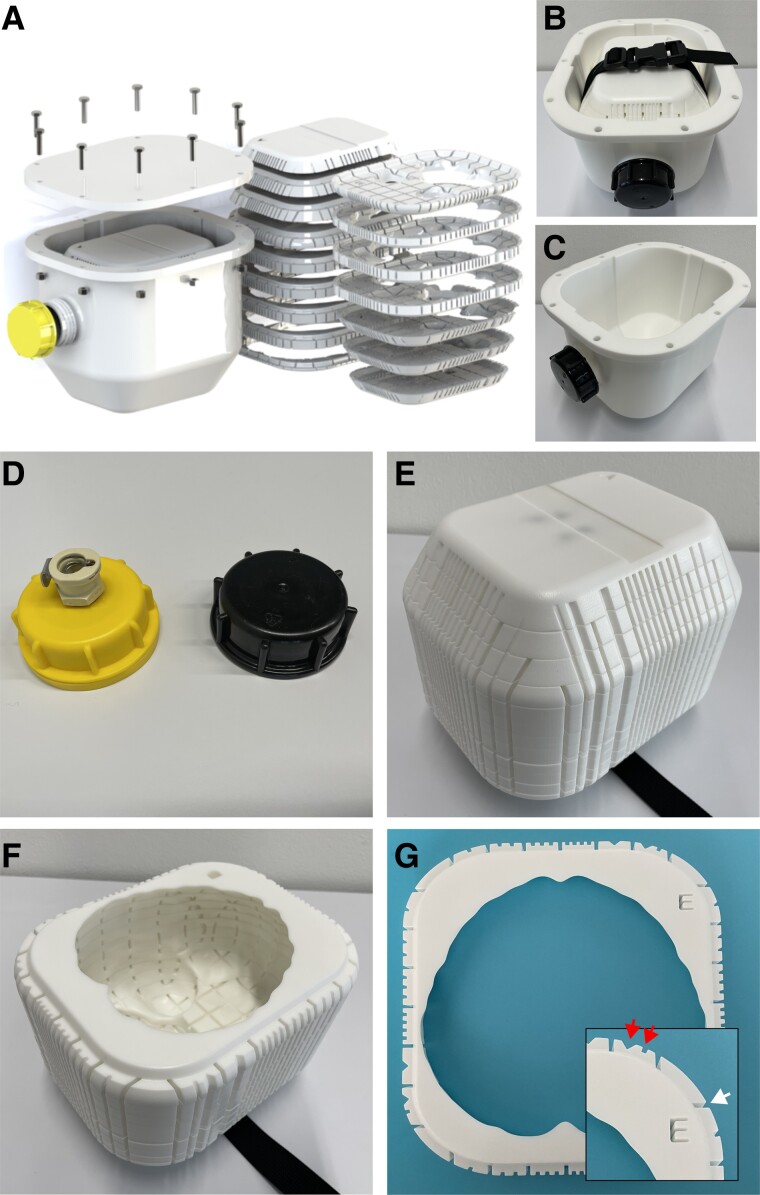
**Brainbox setup.** The MRI-compatible Brainbox: exploded (**A**) and assembled view (**B**). Titanium screws are used to seal the lid. The Brainbox comprises two main parts: first, a watertight tank (outer compartment, (**C**) with an aperture at the front end, for filling or emptying the tank with medium, e.g. water or formalin. The aperture can either be closed using a lid with a vacuum valve for degassing of the specimen (not MRI-compatible) or a standard MRI-compatible lid (**D**). The second part is an inlet holding the brain (brain holder, inner compartment, **E** and **F**). The brain holder can be inserted or removed from the thank with help of a plastic belt with a buckle. This brain holder consists of 16 stackable elements arranged in axial orientation, each with standardized thickness and with an imprinted 3-D coordinate system, identifiable on each individual element (**G**, with inlet showing a higher magnification). The elements have countable and uniquely identifiable indents in X and Y direction (top/red arrows) as well as a Roman letter in Z direction. The elements also have guiding grooves for facilitating cutting using a brain knife (bottom/white arrow). Abbreviations: MRI, magnetic resonance imaging.

Over a period of around four years, the Brainbox was developed as a compoundable, waterproof, and fully MRI-compatible container with an integrated 3D coordinate system. In this paper, we present the most recent fit-for-purpose iteration which is now used in our clinical routine (Fig. 1). Of note, we also include data from the second most recent Brainbox version with a slightly different 3D coordinate system.

To create the Brainbox, a mould was designed based on an averaged brain model, which was inferred from 109 full brain *in vivo* MRI scans, expanded to each side by 1.5 cm. The box was then printed using an HP Jet Fusion 4200 3D printer with a nylon powder print component (HP PA12). Each axially oriented element of the Brainbox was imprinted with a 3D coordinate system to facilitate precise spatial orientation of the brain during and after imaging for correlation with histology features. Note that the imprinted coordinate system allows unequivocal identification of the right and left, anterior and posterior as well as inferior and superior side of the brain and is thus compatible with different spatial axes nomenclatures such as right anterior superior or left anterior superior in the MRI viewer.

Two lids were manufactured for the Brainbox, one with an MR-compatible standard lid and another with a (non-MRI compatible) vacuum valve for degassing the specimen before imaging. The latter lid is replaced with the standard lid after degassing and before magnetic resonance (MR) imaging. Degassing was performed using a benchtop laboratory aspirator system connected to the Brainbox via the vacuum valve and with a vacuum strength of −0.75 bar.

### Human brains and ethical approval

All experimental procedures involving human tissue have been approved by the local ethics board (Cantonal Ethics Committee Zurich, No. 2014-0243) and informed consent has been given by all subjects or their next of kins. Human brains were placed in the Brainbox after autopsy and immersed in phosphate-buffered saline (PBS). The brains were scanned within 3–10 hours after immersion in PBS. Post-mortem intervals ranged from death to brain retrieval between 2 and 18 hours, and from autopsy to scanning between 2 and 18 hours (*Table 1*). After imaging, PBS in the Brainbox was replaced by a 4% formaldehyde solution to fixate the tissue for at least 2 weeks. The brain was then scanned a second time to enable a direct comparison of fresh and formaldehyde-fixed brains. Subsequently, the brains were subjected to autopsy by a neuropathologist. The brains were cut in axial slabs guided by the grooves imprinted on the axial Brainbox elements. Selected imaging features were identified on post- (and if available pre-)mortem MRI scans, and corresponding brain tissue blocks were sampled using a brain knife based on the built-in guidance grooves.

**Table 1:** Cases scanned and processed using the Brainbox

Case no.	Post-mortem intervals (death to scanning)	Age, sex, diagnosis	Pre-mortem MRI available	Fixation times in formaldehyde
1	Unknown^a^	71, F, multiple brain metastases (primary tumour unknown)	No	> 3 years
2	Unknown^a^	62, M, subarachnoid bleeding	Yes	> 3 years
3	4 hours	76, M, embolic strokes in several vascular territories caused by aortic dissection	Yes	2 months
4	36 hours	46, M, X-ALD	Yes	2 months
5	12 hours	65, M, COVID-19	Yes	2 months
6	<12 hours	F, MS	Yes	2 weeks
7	10 hours	14, M, asphyxiation, cavernous haemangioma	No	1 month
8	8 hours	7, F, Unclear enlargement of ventricles with diffuse white matter lesions	No	1 month

Age unknown for the MS patient.

^a^These brains were excluded from the pre- versus post-formaldehyde fixation volume analysis because no pre-fixation MRI was available.

Abbreviations: F, female; M, male; MRI, magnetic resonance imaging; X-ALD, X-linked adrenoleukodystrophy.

### Magnetic resonance imaging

Image acquisition was performed on a 3 Tesla MRI scanner (Magnetom Skyra, Siemens Healthcare, Erlangen, Germany). All MRI images were acquired using a 20-channel head coil for signal reception.

The MRI protocol comprised a T1-weighted (T1w) scout sequence for planning, a high-resolution 3D MPRAGE T1w sequence, a 3D high-resolution T2w SPACE sequence, a 3D high-resolution susceptibility-weighted sequence (SWI), and a 3D high-resolution double inversion recovery (DIR) sequence. All images had a nominal resolution of 0.4 × 0.4 × 0.7 mm^3^ (except the DIR which had a resolution of 0.5 × 0.5 × 0.7 mm^3^), which was sufficient to correlate even small pathologies like a lacunar stroke. Total scanning time was around 2 hours. The sequence parameters are summarized in Supplementary Table 1.

Of note, chemical preservation of post-mortem tissue with aldehyde solutions (e.g. formalin) results in shortened relaxation time constants (T1, T2, and T2*).^22^ This is particularly important for the convergence of T1 of grey and white matter, leading to poor contrast with conventional T1w structural sequences.^23^

MRI volumes of brains before and after formaldehyde fixation were manually segmented on T1-weighted scans using ITK-SNAP.^24^

### Histopathology

Tissue blocks were paraffin-embedded and cut on a microtome into 3–5 µm thick sections. For histological characterization of the MRI features, the following antibodies were used: CD68 (DAKO, clone PG-M1, 1:100), and GFAP (Dako, GA52461-2, 1:100). For immunohistochemistry, slides were incubated with PBS before and blocking in PBS/10% bovine serum albumin for 1 hour at room temperature. Endogenous peroxidase was blocked with 0.3% hydrogen peroxide for 20 min. Slides were incubated with primary antibodies overnight at 4°C. Secondary biotinylated antibody was applied for 1 hour at room temperature followed by the ABC complex reagent (VectorLabs) for one hour. The colour reaction was carried out with ‘ImpactDAB’ (VectorLabs). All counterstainings were done with Haematoxylin. In addition, Luxol Fast Blue staining was used to assess myelination, Prussian Blue for hemosiderin, and Elastica van Gieson staining to highlight elastic lamina in vessel walls. Subsequently, slides were dehydrated in ethanol/xylol and mounted using ‘Entellan’ (Merck Millipore). Slides were scanned using a Hamamatsu slide scanner (Hamamatsu Photonics). Corresponding microphotographs of regions of interest were taken using NDP.view2 viewing software.

### Brainbox MRI-histopathology pipeline

During the common case round, both the post-mortem MRI findings and histopathology findings are discussed by a multidisciplinary team comprising radiologists, neuropathologists and forensic pathologists.

### Statistical analysis

Statistical analysis was conducted in the R programming environment to compare brain volumes before and after formaldehyde fixation (paired *t*-test). The Shapiro-Wilk test was used to assess the normal distribution of our samples. The correlation of pre- and post-fixation volumes was assessed using Pearson’s correlation coefficient.

## Results

### Brainbox

The Brainbox consists of two compartments: a watertight tank (outer compartment, Fig. 1A–C) and an inlet holding the brain (inner compartment or brainholder, Fig. 1A, E, and F). The outer compartment has a size of 23.7 × 20.0 × 19.0 cm, allowing it to fit into a standard 20-channel headcoil from Siemens Skyra. The tank is closed with an MRI-compatible lid using titanium screws and has an aperture at the front end for filling or emptying the tank with fluid. For degassing, a lid with a built-in vacuum valve (not MRI-compatible, Fig. 1D) is used, which is replaced with a standard MRI-compatible lid before imaging.

The brainholder is composed of 16 stackable elements arranged in axial orientation (Fig. 1A, E, and F), and each element has a height/thickness of 15 mm, with varying length and width (15.9–19.6 cm and 10.5–14.5 cm Fig. 1G). The brainholder has a 3D coordinate system with the Z coordinate indicated using Roman letters and the X and Y coordinates using countable indents on the outer surface of the brainholder (Fig. 1G). Each layer is divided into 30–72 quadrants with guidance axes, defining individual brain subvolumes, which can be selected based on imaging volumes of interest. The quadrants have a minimum size of 1.5–2.0 cm, making the excised brain blocks compatible with standard microscope slides. The brainholder can be inserted and removed from the tank using a belt and buckle (Fig. 1B).

### Magnetic resonance imaging

**Figure 2:**
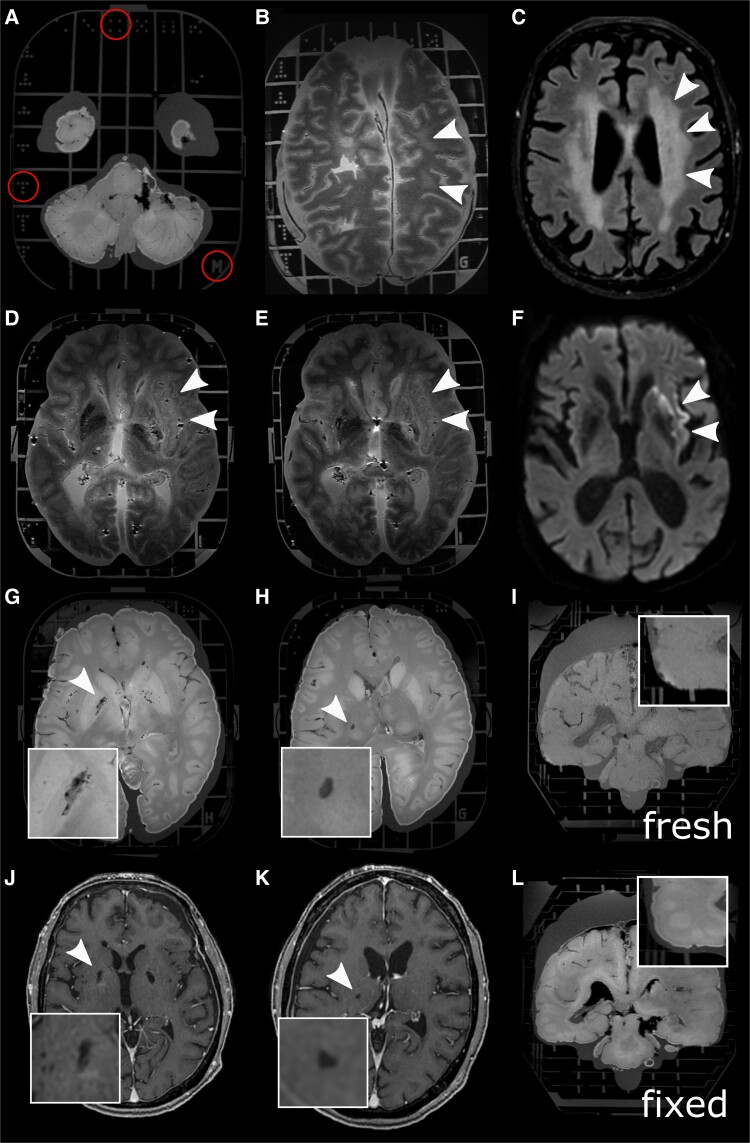
**Whole-brain MRI using the Brainbox.** the Brainbox has an imprinted 3D coordinate system with Latin letters in Z direction and unequivocal dots in X and Y direction (**A**). Whole-brain post-mortem MRI from a patient with X-linked adrenoleukodystrophy shows T2w hyperintensities in the deep white matter (**B**, white arrowheads), similar to the premortem T2w imaging (**C**). T2w hyperintense, i.e. demarcated stroke in the vascular territory of the middle cerebral artery affecting the basal ganglia, the internal capsule and the insula, in fresh (**D**, white arrowheads) and after 3 weeks of formaldehyde fixation (**E**), similar to the diffusion restriction in premortem MRI (**F**). Also, smaller imaging features such as EPVS in the basal ganglia (**G** and **J**, premortem T1w image) or a lacunar stroke (**H** and **K**, premortem T1w scan) are conspicuous using our high-resolution MRI protocol of the whole human brain in the Brainbox (inlets with higher magnification). Brains which were fixed for 2–3 weeks show volume decrease (inlets with higher magnification of the temporal region) (**I** and **L**). Abbreviations: MRI, magnetic resonance imaging; T1w, T1-weighted; T2; T2-weighted.

The study included the scanning of eight human brains using the Brainbox (Table 1). The imprinted 3D coordinate system on the Brainbox allowed the identification of volumes of interest for subsequent correlation to histopathology (Fig. 2A).

**Figure 3:**
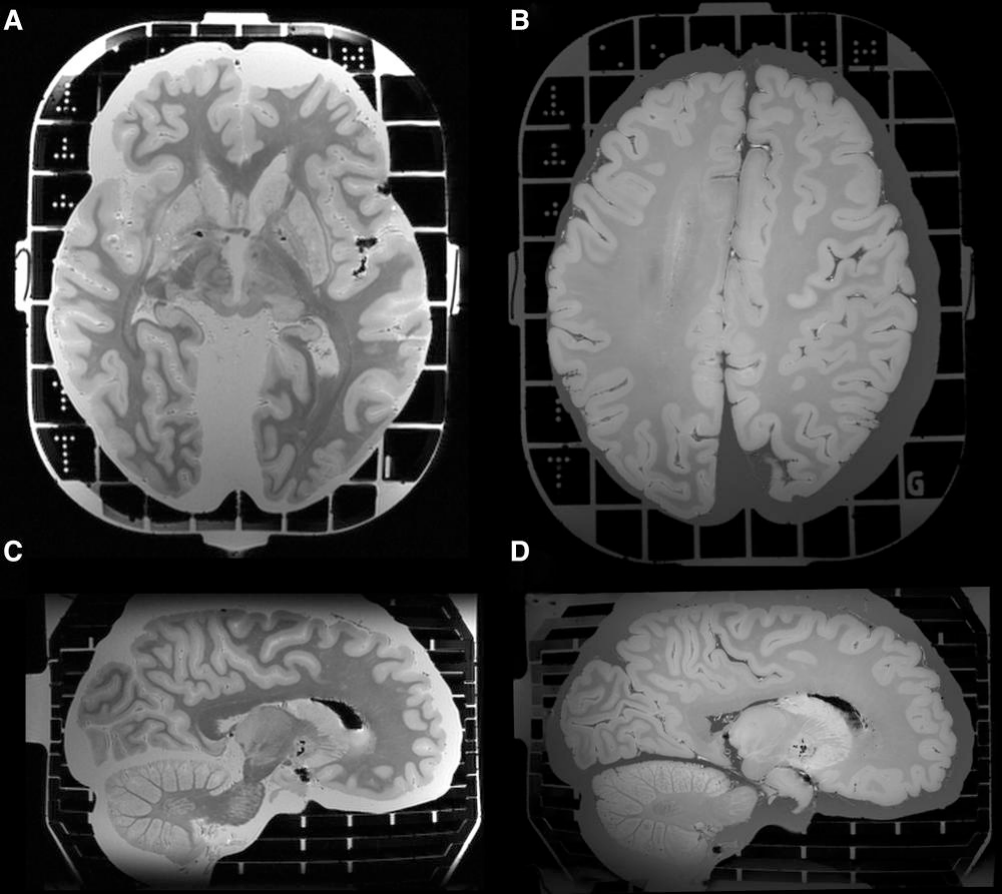
**High-resolution brain MRI from a COVID-19 donor using the Brainbox.** High-resolution MRI of a brain from a COVID-19 donor, employing gradient echo magnetization transfer imaging (**A** and **C**) and gradient echo quantitative susceptibility mapping (**B** and **D**). Nominal resolution: 0.4 mm isotropic.

Whole-brain MRI employing the Brainbox enabled visualization of pathology such as deep white matter T2w-hyperintensities in the parietooccipital lobes in a patient with X-linked adrenoleukodystrophy (X-ALD, Fig. 2B) or a patient with middle cerebral artery stroke (Fig. 2D), similar to the exhibited pathology in premortem MRI (Fig. 2C and F, respectively). The stroke, represented by T2w hyperintensity, was equally well visible in fresh and fixed brain tissue (Fig. 2D and E). The Brainbox was also used to acquire a high-resolution MRI from a COVID-19 donor brain (Fig. 3).

In addition, the Brainbox was able to visualize more discreet and/or confined imaging pathology such as EPVS in the basal ganglia (Fig. 2G) or lacunar strokes (Fig. 2H), similar to the premortem MRI (Fig. J and K, respectively).

Whole brains show a mean volume loss of 7% (standard deviation, ±SD 1%) upon fixation in formaldehyde (fresh: 1200 ml ± SD 69 ml; fixed: 1114 ml ± SD 47 ml, paired *t*-test: *P* = 0.01, 6 brains included in the analysis, Table 1) (Fig. 2I and L, respectively). There was a high correlation between pre- and post-fixation brains volumes (Pearson’s correlation coefficient: 0.64, *P* < 0.01).

We encountered three issues while performing imaging in the Brainbox. (i) The first problem was the presence of air bubbles, which can interfere with the image quality in imaging sequences that are vulnerable to susceptibility artefacts such as T2*w imaging or SWI. Although degassing helped remove most of the air bubbles at the brain surface, it was sometimes necessary to puncture the ventricles using a cannula to release air from the ventricular system. (ii) The second issue we encountered was heating of the tissue/imaging medium during imaging. However, we were able to minimize this problem as our imaging protocol did not result in significant heating of the tissue, resulting in less than 1°C of tissue heating. (iii) The third issue we encountered was ribbon-shaped boundary artefacts that could occur when the brain underwent relatively short fixation before imaging. This artefact resulted in a ribbon-shaped boundary in signal intensity at the grey-white matter junction.

### Correlating MRI features to histopathology/-morphology

The post-mortem MRI scans of the brains were used to identify volumes of interest for correlation of imaging features to their corresponding histopathology. A total of 12 pathomorphological features were correlated, including deep white matter hyperintensities in X-ALD (x3), EPVS in the basal ganglia (x3), cerebellar microbleeds (x2), hippocampi (x2), a lacunar stroke in the thalamus/basal ganglia (x1), and an unclear vascular malformation (x1).

**Figure 4:**
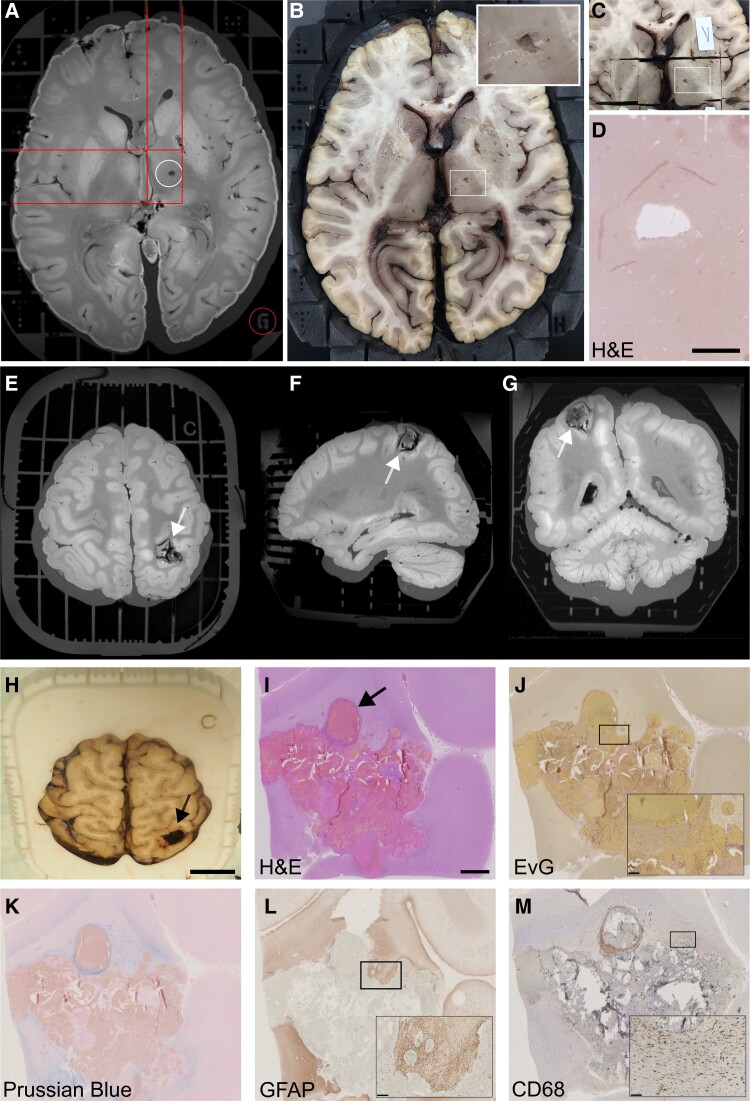
**Imaging to histopathology correlation using the Brainbox.** The Brainbox can facilitate the correlation of MRI features to histopathology. (**A**) A VOI is identified on the MR image, e.g. a hypointense ovoid lesion in the right internal capsule, corresponding to either an enlarged perivascular space (EPVS) or a lacunar stroke (top/white circle). The 3D coordinate system is used to identify its location in relation to the Brainbox (red lines and bottom/red circle). (**B**) Identification of the lesion in the macroscopic view of the brain using the coordinate system (white rectangle labelling VOI, close-up in the top right corner). (**C**) Cutting a tissue block. (**D**) Histopathological work-up using H&E staining and showing a tissue defect, consistent with a chronic lacunar stroke (scale bar: 2 mm). (**E-G**) Unclear haemorrhagic lesion in the right angular gyrus (white arrow). Note the ribbon-shaped artefact at the grey-white matter junction can stem from relatively short brain fixation in formaldehyde. (**H**) This unclear lesion was correlated to its tissue substrate using the Brainbox (scale bar: 2 cm). (**I**) Histopathology using H&E staining reveals a cavernous haemangioma with abnormal blood vessels (black arrow, scale bar: 2 mm). (**J**) Elastica van Gieson (EvG) stain highlights cavernous dilatations and thickened wall in places (inset, scale bar: 250 µm) (**K**) Iron staining using Prussian blue demonstrates hemosiderin in perilesional old micro-haemorrhages (scale bar: 250 µm). (**L** and **M**) Immunohistochemistry demonstrates fibrillary GFAP-positive astrogliosis (**L**) and CD68 in reactive microglia (**M**) (scale bar: **A**: 2 cm, I-M 2 mm, insets: 250 µm). Abbreviations: EvG, Elastica van Gieson; GFAP, glial fibrillary acidic protein; H&E, haematoxylin and eosin; MRI, magnetic resonance imaging.

With its 3D coordinate system, the Brainbox enabled accurate correlation of imaging features to histopathology. This is demonstrated by two concrete examples of MRI-histopathology correlation: (i) A T1w hypointense ovoid lesion (2 × 3 mm) in the right internal capsule most likely representing an enlarged perivascular space or a chronic lacunar stroke (Fig. 4A). This MRI finding was defined using the 3D coordinate system of the Brainbox (Fig. 4A). Subsequently, respective coordinates were identified on the Brainbox by disassembling the axially arranged elements until the eligible Z coordinate was located. Using the axially oriented guidance cue of the Brainbox, the brain was dissected in axial direction using a brain knife (Fig. 4B). With this, the volume of interest (VOI) became apparent within the brain. Then, the corresponding guidance grooves in X and Y direction were used to dissect the brain tissue block of interest (Fig. 4C). These brain blocks were then histopathologically processed. Analysis of respective tissue sections showed a focal tissue defect on the Haematoxylin and Eosin staining, a finding consistent with a lacunar stroke (Fig. 4D). (ii) By employing a similar approach as for the lacunar stroke, we used the Brainbox during autopsy workup of a case from the forensic medicine: We correlated an unclear MRI lesion with susceptibility artefacts in the right angular gyrus (Fig. 4E–G), likely responsible for a fatal generalized seizure. The lesion was identified in the brain using the 3D coordinate system of the Brainbox (Fig. 4H). The histopathological workup of corresponding brain tissue blocks showed abnormal blood vessels with cavernous dilatations and irregularly thickened walls consistent with a cavernous haemangioma surrounded by reactive gliosis (Fig. 4I–M).

**Figure 5:**
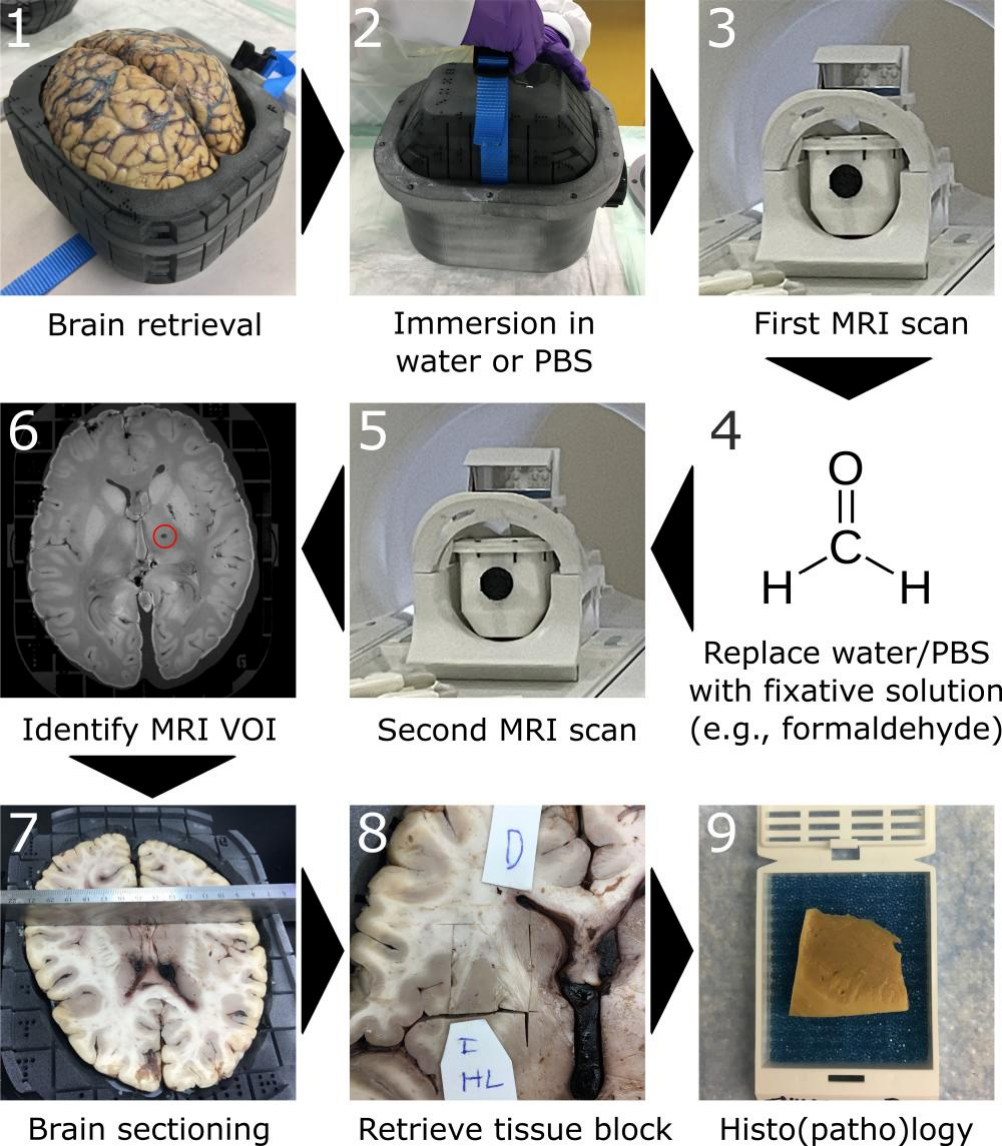
**Pipeline for MRI-histopathology correlation using the Brainbox.** 1: The brain is retrieved upon autopsy and mounted in the brain holder (inner compartment) of the Brainbox; 2: The brain holder is inserted into the tank filled with an aqueous medium, e.g. water or PBS; 3: First MRI scan of the brain using a 20-channel head coil; 4: Replace aqueous medium with fixative solution, e.g. 10% neutral-buffered formalin. Of note, the medium can be changed by pouring out the medium and filling the tank with another medium, i.e. the brain position within the brain holder remains unchanged. The brain can be soaked/stored until acquisition of next MRI; 5: Second MRI scan; 6: Identification of a VOI within the MR images, e.g. a presumable lacunar stroke. Reference points for later tissue retrieval can be identified on the built-in 3D coordinate system; 7: Axial/transversal sectioning of the brain according to the identified coordinates; 8: Retrieval of tissue block of interest; 9: Histo(patho)logical processing of tissue block, e.g. embedding in paraffin. Abbreviations: MRI, magnetic resonance imaging; PBS, phosphate-buffered saline; VOI, volume of interest.

During the common case round, both the post-mortem MRI findings and histopathology findings are discussed by a multidisciplinary team comprising radiologists, neuropathologists, and forensic pathologists. The pipeline is shown in Fig. 5. This collaborative approach has improved the accuracy of diagnoses and facilitated the identification of findings that might have been missed by either department alone, for example cerebellar bleeding or an unclear lesion in the angular gyrus (Fig. 4E–M).

## Discussion

### Main findings

We herein introduce the Brainbox, a tool that facilitates the correlation of post-mortem *ex vivo* MRI and histopathology of human brains. Its 3D coordinate system enabled unequivocal identification of MRI features of interest and correlation to corresponding tissue features. The Brainbox minimized susceptibility artefacts and bulk motion during scanning. The tool has been validated in eight human brains. The Brainbox has facilitated collaboration between the departments of neuroradiology, neuropathology and forensic medicine through a common case round.

### Findings in the context of existing evidence

*Ex vivo* imaging studies are commonly done on brain slices or hemispheres. However, imaging the whole brain has several advantages, among them fewer artefacts (e.g. air bubbles and distortion edges) and reduced total scan time for covering the whole brain. However, most importantly, whole-brain imaging preserves landmarks for radiologic interpretation which is especially important for exploratory imaging or examining small focal and more diffuse neuropathologic features. These reasons render whole-brain imaging the most practical approach for clinical applications.^25^ Moreover, whole-brain imaging is essential for detecting subtle pathology along white matter tracts with advanced techniques such as tractography. With this, we have established a collaboration with the neuropathology and forensic medicine at our hospital for work-up of cases using the Brainbox in clinical rounds.

Commonly faced problems during *ex vivo* imaging are air bubbles and (bulk) motion of the brain within the container during scanning caused by buoyancy and/or vibration by certain MRI sequences. The presence of air bubbles can interfere with the image quality in imaging sequences that are vulnerable to susceptibility artefacts such as T2*w imaging or SWI. Although degassing helped remove most of the air bubbles at the brain surface, it was sometimes necessary to puncture the ventricles using a cannula to release air from the ventricular system. Other dedicated approaches to minimize susceptibility artefacts by removing air bubbles from the specimen have been developed.^26,27^ Also, bulk motion was reduced by our Brainbox by serving as a rigid scaffold holding the brain during scanning. Other solutions mitigating bulk motion have been proposed, e.g. by a Plexiglas container which minimizes bulk motion of the brain during scanning.^28^ We encountered additional challenges while acquiring MR images using the Brainbox: (i) Heating of the tissue/imaging medium during imaging. We were able to minimize this problem as our imaging protocol did not result in significant heating of the tissue, resulting in less than 1°C of heating; and (ii) Ribbon-shaped boundary artefacts that occurred when the brain was subjected to a relatively short fixation period before imaging. This artefact resulted in a ribbon-shaped boundary in signal intensity at the grey-white matter junction. Note that immersion fixation can also lead to gradients in fixation quality regardless of fixation time. This is because more superficial brain regions undergo superior tissue preservation compared to deeper brain regions.^29^

Other methods for correlating MRI features to the tissue substrate have been developed. Lasserve and colleagues have introduced a 3D-printed mould mounting a whole brain (or a medial temporal lobe) with interactively placeable guiding indents for sectioning.^30,31^ Another approach has been developed by Absinta/Guy and colleagues: an individualized, 3D printed cutting box for human^32^ or marmoset brains.^33^ This method enables monoplanar sectioning of the brain subsequently to post-mortem imaging to improve alignment of post-mortem MRI and autopsy brain slabs. Although these approaches are undoubtfully valuable, they are employed after MRI acquisition. In contrast, the Brainbox contains the brain during the entire imaging session as well as the subsequent brain sectioning which alleviates correlation of MRI features to histopathology. With this, the Brainbox can also be used for longterm storage and transport of a whole human brain. It enables time savings in a similar range like published devices,^32^ i.e. around 2 hours versus 10–15 hours for manual correlation. Of note, while it is recommended to preserve samples under appropriate conditions to maintain their integrity, short-term storage at room temperature for neuroimaging in the Brainbox may have minimal impact on commonly used ‘omics’ analyses.^34,35^

Post-mortem MRI is an intermediate step for understanding the pathologic basis for MRI signal changes. Yet care should be taken when inferring findings from *ex vivo* histopathology correlation to the *in vivo* paradigm. Findings from a unique case report acquiring ante-mortem and *in situ* post-mortem MRI within only a 4-day interval suggested that brain volume, particularly in the white matter, can substantially increase upon death.^36^ Additional alterations are entailed by tissue processing before autopsy, mostly by formaldehyde-based fixation agents. Such fixation agents change tissue T1 and, to a lesser degree, T2 by decreasing the difference in water mobility between grey and white matter.^22,37–42^ Early decline in T1 during fixation can lead to convergence of T1 in both grey and white matter^43^ resulting in poorer grey-white matter contrast or even reversal of relative signal intensities between grey and white matter in T1w sequences.^23,28,44^ Stable changes of T1 and T2 can be expected after 3–4 weeks for brain tissue samples and a few months for whole brains.^43^ Based on this paradigm, dedicated MRI sequences such as spin (proton) density-weighted protocols^28^ or steady-state based sequences (e.g. steady-state free precession or TrueFISP) can be advantageous for *ex vivo* MRI.^7^ Of note, long post-mortem intervals can be a confounding factor because they can be associated with tissue decomposition.^45,46^

For most clinical scenarios where our Brainbox could be employed, a qualitative correlation of MRI to histopathology will be sufficient. However, certain research questions might require a more accurate correlation of these two domains. In such a case, the histologically stained section can be registered to the *ex vivo* MRI.^42^ This process can be challenging due to nonlinear tissue deformation caused by histopathological processing, e.g. tissue sectioning, slide-mounting, air drying, or treatment with solvents.^47–52^ This challenge is particularly relevant for human brain tissue with its highly convoluted cortex. Accurate registration warrants at least a prior affine registration and subsequent deformable alignment.^47,50,53–56^ Blockface images, i.e. images from tissue blocks obtained during sectioning, can facilitate registration by allowing MRI-to-block registration as intermediate step.^42^

### Limitations

Our Brainbox has some limitations to consider. First, Fomblin, a chemically inert perfluoropolyether fluorocarbon which yields no MRI signal and has a similar magnetic susceptibility to tissue, is commonly used for *ex vivo* MR imaging.^23,32,57^ Currently, the integrated 3D-coordinate system of the Brainbox, which is visualized using the MR signal of an aqueous medium, renders the use of Fomblin suboptimal. However, of note, Fomblin is expensive (>500 US$ per litre) and leaves oily remnants on the tissue surface which may interfere with subsequent histopathological work-up.^26^ Second, although degassing helped to reduce air bubbles, small remaining air bubbles in the ventricles were commonly encountered. A solution for this has been proposed by Shatil and colleagues, i.e. to inject liquid into the ventricles using a syringe.^26^ Yet neuropathologists should be consulted in advance to avoid interference with the autopsy. Third, the default method for brain sectioning using our Brainbox is the axial/transversal plane which makes the handling of the Brainbox more viable and which is the standard plane for brain MRI scans. This contrasts the coronal sectioning as the standard cutting plane for autopsy. Future iterations of the Brainbox will enable coronal sectioning. Fourth, despite the reliable correlation of imaging features to histopathology, our high-resolution MRI scans were anisotropic. Larger scale projects using the Brainbox could implement isotropic MRI scans as regularly done by the Human Connectome Project.^58^

## Conclusions

The Brainbox cannot only be used for longterm storage and transport of whole human brains but it also facilitates correlation of MRI features to histopathology. With this, our approach embodies the concept that whole-brain *ex vivo* MRI can alleviate and guide neuropathology thus providing a benchmark for comparison before and after sectioning. This can ultimately benefit the specificity of MRI. Brainboxes are available upon request from the corresponding authors.

## Supplementary Material

fcad307_Supplementary_Data

## Data Availability

The MRI data used to investigate our research questions are only available upon special request and in accordance with current legislation since these are sensitive patient data. The R code to conduct the statistical analysis is available upon request from the corresponding author. Brainboxes are available upon request from the corresponding authors.
