# Supplementary material for: The Brainbox—a tool to facilitate correlation of brain magnetic resonance imaging features to histopathology
Source: Brain Commun. 2023 Nov 8;5(6):fcad307. doi: 10.1093/braincomms/fcad307 (PMC10664401; doi:10.1093/braincomms/fcad307)
Supplement: fcad307_Supplementary_Data [file fcad307_supplementary_data.pdf]

Supplementary data

Supplementary Table 1: Sequence parameters for 3D MRI protocol

Sequence	TE (ms)	TR (ms)	Flip angle	FOV	Acquisition time (min)	Nominal resolution (mm)
T1w MPRAGE	5.85	4160	15	256	32:12	0.4 x 0.4 x 0.7
T2w SPACE	317	2000	N/A	204	31:14	0.4 x 0.4 x 0.7
SWI	20	50	20	230	43:04	0.4 x 0.4 x 0.7
DIR	322	7500	N/A	204	14:54	0.5 x 0.5 x 0.7

Glossary: DIR, double inversion recovery; FOV, field of view; ms, milliseconds; SWI, susceptibility weighted imaging; TE, echo time; TR, repetition time.
